# Murine model identifies tropomyosin as IgE cross-reactive protein between house dust mite and coho salmon that possibly contributes to the development of salmon allergy

**DOI:** 10.3389/fimmu.2023.1238297

**Published:** 2023-08-30

**Authors:** Risa Yamamoto, Kumi Izawa, Tomoaki Ando, Ayako Kaitani, Atsushi Tanabe, Hiromichi Yamada, Shino Uchida, Akihisa Yoshikawa, Yasuharu Kume, Shun Toriumi, Akie Maehara, Hexing Wang, Masakazu Nagamine, Naoko Negishi, Nobuhiro Nakano, Nobuyuki Ebihara, Toshiaki Shimizu, Hideoki Ogawa, Ko Okumura, Jiro Kitaura

**Affiliations:** ^1^ Atopy (Allergy) Research Center, Juntendo University Graduate School of Medicine, Tokyo, Japan; ^2^ Department of Pediatrics and Adolescent Medicine, Juntendo University Graduate School of Medicine, Tokyo, Japan; ^3^ Department of Gastroenterology, Juntendo University Graduate School of Medicine, Tokyo, Japan; ^4^ Department of Otorhinolaryngology, Juntendo University Graduate School of Medicine, Bunkyo, Tokyo, Japan; ^5^ Department of Ophthalmology, Juntendo University Urayasu Hospital, Urayasu, Chiba, Japan; ^6^ Department of Science of Allergy and Inflammation, Juntendo University Graduate School of Medicine, Bunkyo, Tokyo, Japan

**Keywords:** food allergy, IgE cross-reactivity, house dust mite, salmon, mast cell, murine model

## Abstract

**Background:**

Recently, we have developed a method to identify IgE cross-reactive allergens. However, the mechanism by which IgE cross-reactive allergens cause food allergy is not yet fully understood how. In this study, we aimed to understand the underlying pathogenesis by identifying food allergens that cross-react with house dust mite allergens in a murine model.

**Material and methods:**

Allergenic protein microarray analysis was conducted using serum from mice intraperitoneally injected with *Dermatophagoides pteronyssinus* (Der p) extract plus alum or alum alone as controls. Der p, *Dermatophagoides farinae* (Der f), coho salmon extract-sensitized and control mice were analyzed. Serum levels of IgE against Der p, Der f, coho salmon extract, protein fractions of coho salmon extract separated by ammonium sulfate precipitation and anion exchange chromatography, and recombinant coho salmon tropomyosin or actin were measured by an enzyme-linked immunosorbent assay. A murine model of cutaneous anaphylaxis or oral allergy syndrome (OAS) was established in Der p extract-sensitized mice stimulated with coho salmon extract, tropomyosin, or actin.

**Results:**

Protein microarray analysis showed that coho salmon-derived proteins were highly bound to serum IgE in Der p extract-sensitized mice. Serum IgE from Der p or Der f extract-sensitized mice was bound to coho salmon extract, whereas serum IgE from coho salmon extract-sensitized mice was bound to Der p or Der f extract. Analysis of the murine model showed that cutaneous anaphylaxis and oral allergic reaction were evident in Der p extract-sensitized mice stimulated by coho salmon extract. Serum IgE from Der p or Der f extract-sensitized mice was bound strongly to protein fractions separated by anion exchange chromatography of coho salmon proteins precipitated with 50% ammonium sulfate, which massively contained the approximately 38 kDa protein. We found that serum IgE from Der p extract-sensitized mice was bound to recombinant coho salmon tropomyosin. Der p extract-sensitized mice exhibited cutaneous anaphylaxis in response to coho salmon tropomyosin.

**Conclusion:**

Our results showed IgE cross-reactivity of tropomyosin between *Dermatophagoides* and coho salmon which illustrates salmon allergy following sensitization with the house dust mite *Dermatophagoides*. Our method for identifying IgE cross-reactive allergens will help understand the underlying mechanisms of food allergies.

## Introduction

The incidence of allergic diseases, such as food allergies, atopic dermatitis, and allergic rhinitis, is increasing worldwide. Food allergies, including oral allergy syndrome (OAS), are an increasing public health concern. Food allergen-specific immunoglobulin E (IgE) production, also known as IgE sensitization to food allergens, often precedes the development of food allergies. Intake of the same food allergen induces the crosslinking of its specific IgE-bound high-affinity IgE receptor (FcεRI) with the same allergen in mast cells. Consequently, mast cells degranulate and release various chemical mediators, leading to food allergy symptoms, such as diarrhea, abdominal pain, and anaphylaxis in severe cases (1–8). In the case of OAS, mast cell degranulation in the oral and pharyngeal tissues causes itching and/or angioedema of the lip, tongue, and palate. Pollen food allergy syndrome (PFAS) is a pollinosis-associated OAS; IgE cross-reactive allergens between certain foods (e.g., fruits and vegetables) and pollens (e.g., birch and ragweed pollens) play critical roles in the development of PFAS (9–15). Understanding how some people become sensitized to specific food allergens is important for the prevention of food allergies. We recently developed a method for the comprehensive identification of foods that may cross-react with pollen, using murine models of sensitization to pollen and allergic protein microarray technology (15). This encouraged us to apply our method to identify IgE cross-reactivity among various environmental allergens, including food allergens.

House dust mites (HDM) are well-known allergens that contribute to the development of allergic diseases, such as atopic dermatitis, allergic rhinitis, and allergic asthma with specific IgEs against HDM allergens. *Dermatophagoides pteronyssinus* (Der p) and *Dermatophagoides farinae* (Der f) are the most known HDM species and have been reported to include up to 40 distinct allergens (16, 17). Among them, mite tropomyosin Der p 10 or Der f 10 shows IgE cross-reactivity with shrimp tropomyosin, which is thought to be responsible for shrimp allergy (18, 19). However, a causal relationship between HDM and food allergies is not yet fully understood.

Seafood can be classified into three different groups: arthropods, mollusks and vertebrates. Crustaceans (e.g., shrimp and crab) are arthropods, while cephalopods (e.g., squid and octopus) and bivalves (e.g., clams and oysters) are mollusks. Fish (e.g., salmon and tuna) are aquatic vertebrates. Fish allergy is also an IgE-mediated food allergy, and its prevalence has increased, particularly in countries with high fish consumption. Parvalbumin, a fish pan-allergen, is a low-molecular-weight protein abundant in fish muscle. Enolase, aldolase, and gelatin are recognized fish allergens. A recent study using serum from patients with salmon or catfish allergies identified tropomyosin as an important allergen, indicating that tropomyosin, in addition to parvalbumin, should be considered an important fish allergen to improve the diagnosis of fish allergy (20–22).

In this study, we identified coho salmon as a food that may cross-react with Der p extract using a murine model of sensitization to Der p extract and protein microarray technology (15). *In vitro* and *in vivo* analyses demonstrated IgE cross-reactivity between Der p and coho salmon extracts. Tropomyosin was identified as an IgE cross-reactive protein that mediates local anaphylactic reactions in a murine model of cutaneous allergy and OAS. Our method will be useful for clarifying the pathogenesis of food allergies caused by IgE cross-reactivity between environmental proteins.

## Materials and methods

### Mice

Female BALB/c mice (aged 8–12 weeks) were used in this study. All the procedures were approved by the Institutional Review Committee of Juntendo University (approval numbers: 2021188, 2022099, and 2023129).

### Reagents

**Figure 1:**
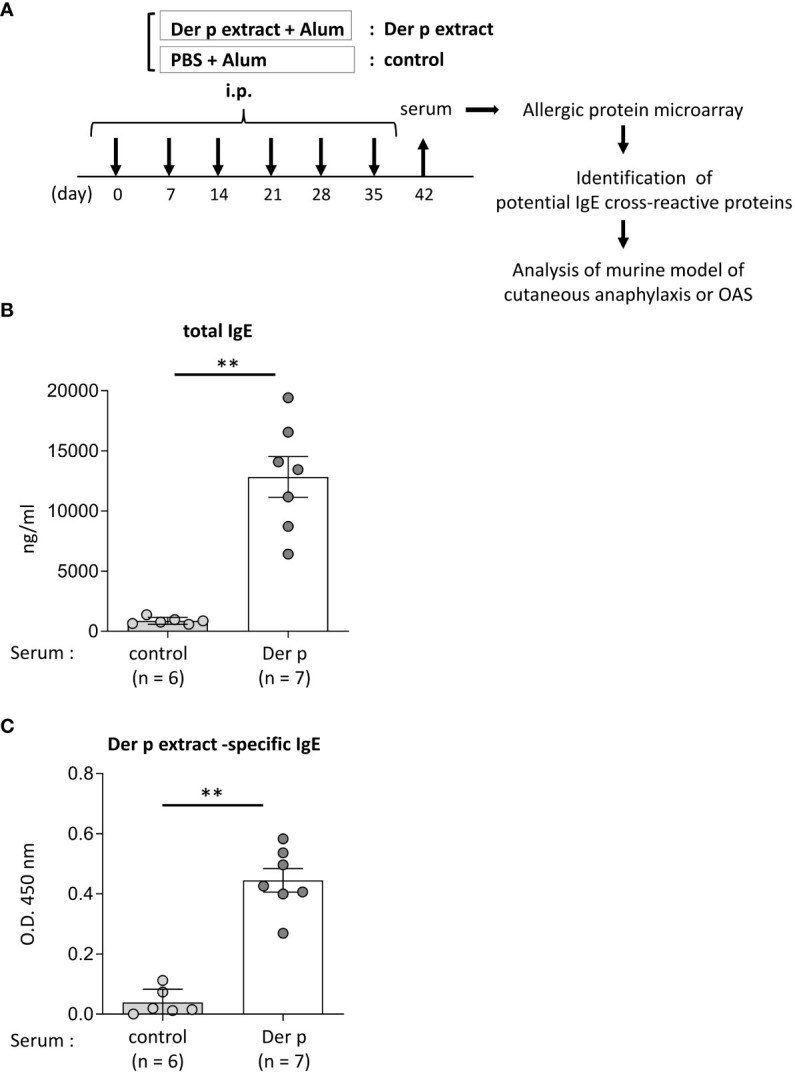
Identification of foods that may cross-react with Der p extract by using a murine model of sensitization with Der p extract and allergenic protein microarray technology.**(A)** Allergenic protein microarray analysis was performed using serum from BALB/c mice that had been intraperitoneally injected with Der p extract plus alum (Der p extract) or PBS plus alum as a control (control) six times at a 1- week interval, prior to analysis of the murine model of cutaneous anaphylaxis or OAS. **(B, C)** Serum levels of total IgE **(B)** and Der p extract-specific IgE **(C)** from mice on day 42. Data are representative of two independent experiments. Means ± SD have been plotted. **P < 0.01.

**Figure 2:**
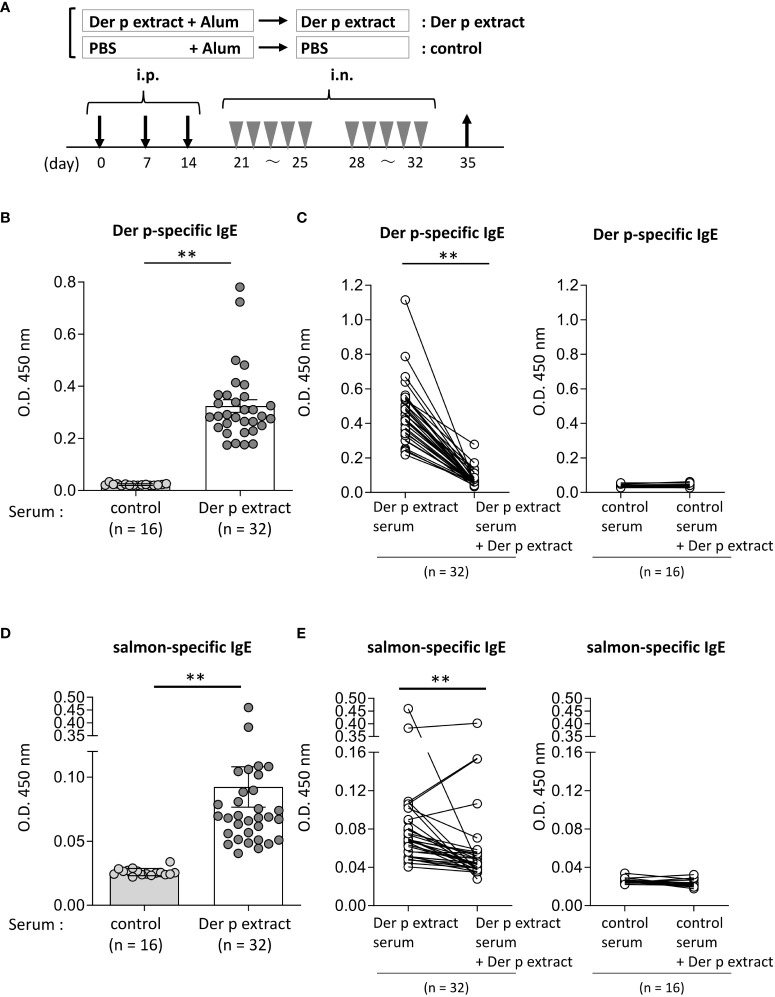
Serum IgE from mice sensitized with the Der p extract was significantly bound to the salmon extract. **(A)** A schematic representation of mouse sensitization with Der p extract. Mice were intraperitoneally injected with Der p extract plus alum or PBS plus alum alone followed by intranasal administration of Der p extract or PBS to generate Der p extract-sensitized mice (Der p extract) or control mice (control), respectively. On day 35, blood samples were obtained. **(B, D)** Serum levels of specific IgE from Der p extract-sensitized mice or control mice against **(B)** Der p extract and **(D)** coho salmon extract. **(C, E)** Serum levels of specific IgE from Der p extract-sensitized mice or control mice against **(D)** Der p extract and **(F)** coho salmon extract after serum preincubation with Der p extract or PBS as a control. **(B–E)** Data were pooled from three independent experiments. Means ± SD have been plotted. **P < 0.01.

**Figure 3:**
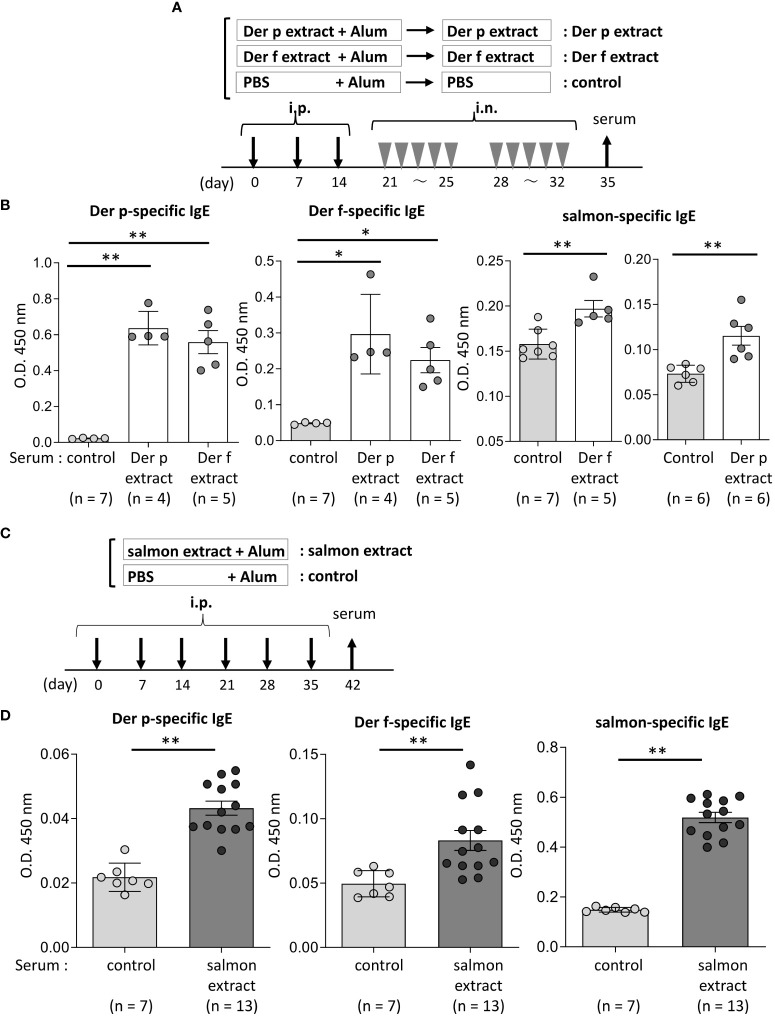
Serum IgE from mice sensitized with coho salmon extract was significantly bound to Der p and Der f extract. **(A)** A schematic representation of mouse sensitization with Der p or Der f extract. Mice were intraperitoneally injected with Der p or Der f extract plus alum or PBS plus alum alone followed by intranasal administration of Der p or Der f extract or PBS to generate Der p or Der f extract-sensitized mice (Der p extract or Der p extract) or control mice (control), respectively. On day 35, blood samples were obtained. **(B)** Serum levels of specific IgE from Der p or Der f extract-sensitized mice or control mice against Der p extract (left), Der f extract (middle), or coho salmon extract (right). **(C)** A schematic representation of mouse sensitization with coho salmon extract. Mice were intraperitoneally injected with coho salmon extract plus alum or PBS plus alum six times at a 1- week interval to generate coho salmon extract-sensitized mice (salmon extract) or control mice (control), respectively. On day 42, blood samples were obtained. **(D)** Serum levels of specific IgE from salmon extract-sensitized mice or control mice against Der p extract (left), Der f extract (middle), or coho salmon extract (right). **(B, D)** Data are representative of two independent experiments. Means ± SD have been plotted. *P < 0.05 or **P < 0.01.

**Figure 4:**
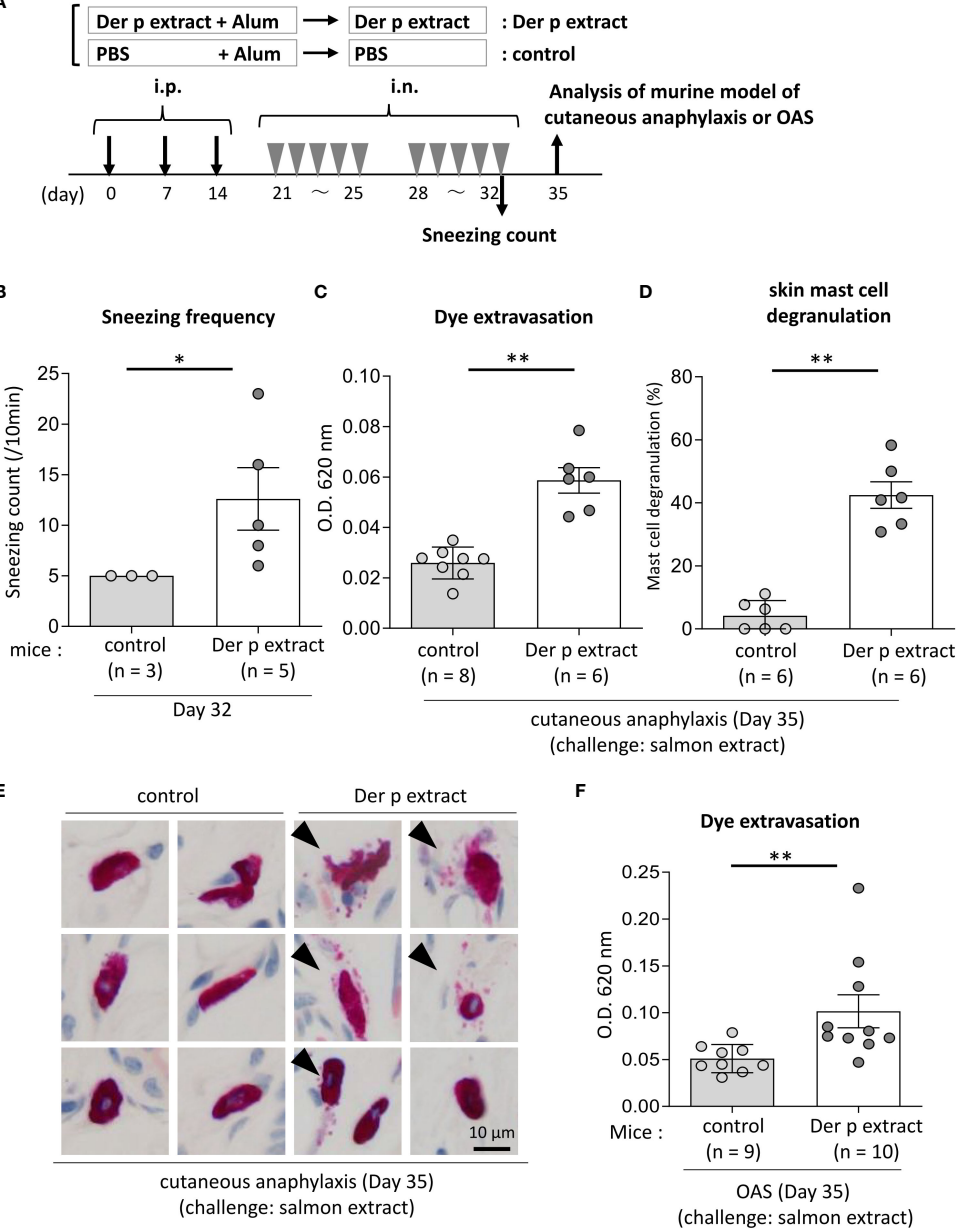
Both cutaneous anaphylaxis and oral allergy were evident in Der p extract-sensitized mice after administration of coho salmon extract. **(A)** A schematic representation of the murine model of cutaneous anaphylaxis or OAS. Mice were intraperitoneally injected with Der p extract plus alum or PBS plus alum followed by intranasal administration of Der p extract or PBS to generate Der p extract-sensitized mice (Der p extract) or control mice (control), respectively. On day 35, mice were challenged with coho salmon extract. **(B)** The frequency of sneezing during 30 min after nasal administration of Der p extract or PBS on day 32. **(C–E)** The ear skin was removed from Der p extract-sensitized mice or control mice stimulated by coho salmon extract in murine model of cutaneous anaphylaxis. **(C)** Quantification of the Evans blue dye. **(D)** Percentages of degranulated mast cells. **(E)** Representative images of chloroacetate esterase-stained mast cells in tissue sections. The arrowhead indicates the degranulated mast cell. **(F)** Quantification of the Evans blue dye that extravasated into the neck skin from Der p extract-sensitized mice or control mice stimulated by coho salmon extract in the murine model of OAS. **(B–F)** Data are representative of two independent experiments. Means ± SD have been plotted. *P < 0.05 or **P < 0.01.

**Figure 5:**
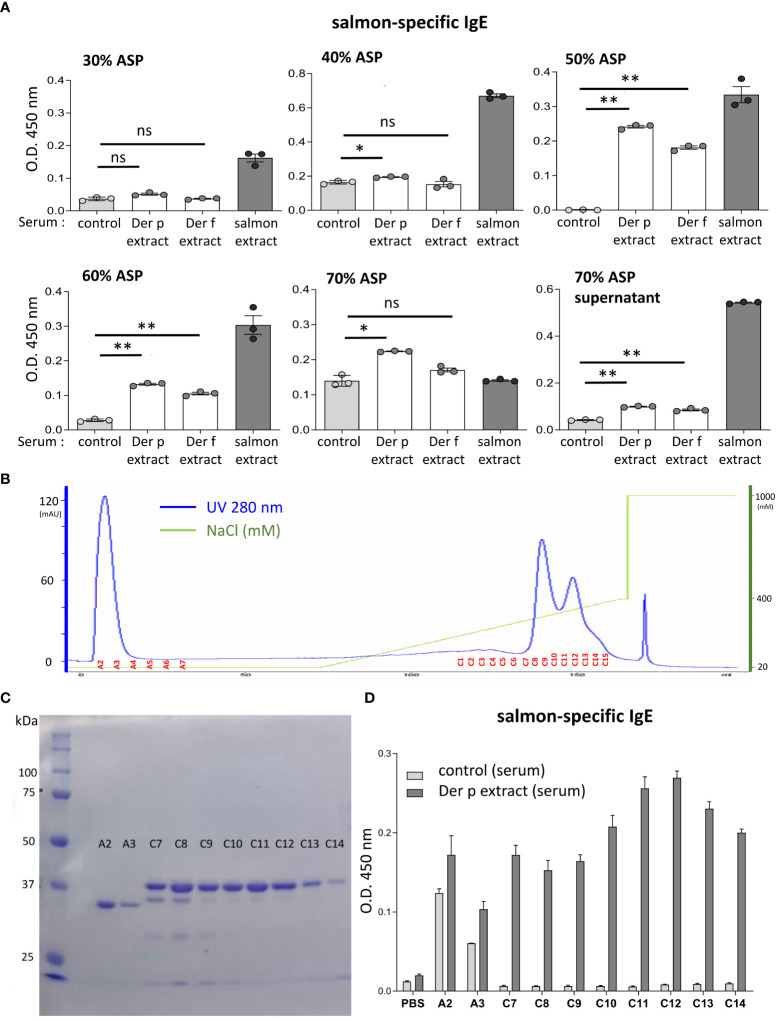
The molecular weight of the approximately 38-kDa protein included in coho salmon extract was possibly recognized by serum IgE from Der p extract-sensitized mice. **(A)** Serum levels of specific IgE from Der p or Der f extract-sensitized mice (Der p extract or Der f extract), control mice (control), or coho salmon extract-sensitized mice (salmon extract) against coho salmon proteins precipitated with 30%, 40%, 50%, 60%, or 70% ammonium sulfate (30% ASP, 40% ASP, 50% ASP, 60% ASP, 70% ASP) or soluble in 70% ammonium sulfate (70% ASP supernatant). **(B)** Coho salmon proteins precipitated with 50% ammonium sulfate were subjected to anion exchange chromatography. Spectra of anion exchange chromatography fractions are shown. The blue line indicates a UV 280 nm signal. The green line indicates NaCl concentration in the buffer. **(C)** Equivalent amounts of total protein in each fraction of A2, A3, and C7 to C14 were subjected to SDS-PAGE before CBB staining. **(D)** Serum levels of specific IgE from Der p extract-sensitized mice or control mice against proteins of each fraction (A2, A3, and C7 to C14), salmon extracts, or vehicle (PBS). **(A–D)** Data are representative of two independent experiments. **(A)** Means ± SD have been plotted. *P < 0.05 or **P < 0.01. ns, not significant.

**Figure 6:**
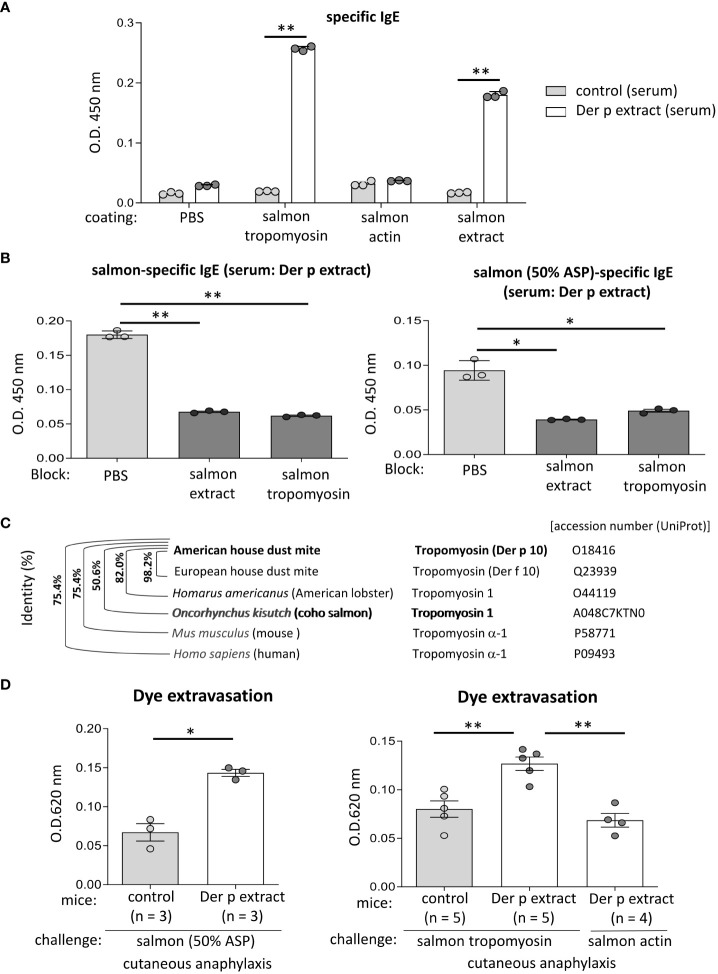
Tropomyosin was identified as IgE cross-reactive protein between Der p and coho salmon extract. **(A)** Serum levels of specific IgE from Der p extract-sensitized mice (Der p extract) or control mice (control) against recombinant coho salmon tropomyosin or actin, coho salmon extracts, or vehicle (PBS). **(B)** Serum levels of specific IgE from Der p extract-sensitized mice after serum preincubation with coho salmon extract, recombinant coho salmon tropomyosin, or PBS. **(C)** Percentages of amino acid sequence identity of Der p 10/tropomyosin from American house dust mite with Der f 10/tropomyosin from European house dust mite, tropomyosin 1 from *Homarus americanus* (American lobster), tropomyosin 1 from *Oncorhynchus kisutch* (coho salmon), tropomyosin α-1 from *Mus musculus* (mouse), or tropomyosin α-1 from *Homo sapiens* (human). The accession number (UniProt) is shown. **(D)** Quantification of the Evans blue dye that extravasated into the ear skin from Der p extract-sensitized mice or control mice stimulated by coho salmon proteins precipitated with 50% ammonium sulfate (left panel) or recombinant coho salmon tropomyosin or actin (right panel) in murine model of cutaneous anaphylaxis. **(A, B, D)** Data are representative of two independent experiments. Means ± SD have been plotted. *P < 0.05 or **P < 0.01.

The Der p extract (Biostir Inc., Kobe, Japan) was used, as shown in **Figures 1**, **2**, **4**. Der f extract (Greer Laboratories, Lenoir, NC) or Der p extract (Greer Laboratories, Lenoir, NC) was used, as shown in **Figures 3**, **5**, **6**. A fillet of commercially available coho salmon in Japan was used to prepare coho salmon (*Oncorhynchus kisutch*) extract.

### Allergenic protein microarray

BALB/c mice were intraperitoneally injected with 30 μg Der p extract plus 2 mg alum or with phosphate-buffered saline (PBS) plus 2 mg alum as a control six times at 1-week intervals to generate Der p extract-sensitized or control mice, respectively. The serum obtained from these mice on day 42 was incubated on microarray plates coated with 1178 types of crude allergenic protein extracts (Fukushima Translational Research Project, Fukushima, Japan), including plants (e.g., vegetables and fruits), animals, processed foods (e.g., fish, shellfish, insects, ticks, parasites, meats, eggs, cheeses, and yogurts), and microorganisms (e.g., bacteria and fungi). *Oncorhynchus kisutch* (coho salmon), *Scomber japonicus* (Chub mackerel), *Gadus macrocephalus* (Pacific cod), *Sardinops melanostictus* (sardine), and *Thunnes orientalis* (Pacific bluefin tuna) were used as fish. Alexa Fluor 647-conjugated anti-mouse IgE antibody was added to the wells before the microarray plates were scanned with a GenePix 4000 B scanner (Molecular Devices, San Jose, CA, USA) to measure fluorescence intensity. The relative binding intensity of serum IgE to each protein extract was calculated by subtracting the IgE binding intensity of control mice from that of the Der p extract-sensitized mice (15, 23).

### Production of recombinant protein of coho salmon tropomyosin or actin

The amino acid sequences of *Oncorhynchus kisutch* (coho salmon) tropomyosin and actin were retrieved from UniProt (tropomyosin 1, accession: A0A8C7KTN0, https://www.uniprot.org/uniprotkb/A0A8C7KTN0/entry; and actin, alpha skeletal muscle 2-like, accession: A0A8C7G2G1, https://www.uniprot.org/uniprotkb/A0A8C7G2G1/entry). The sequences were reverse-translated into codon-optimized DNA sequences for *Escherichia coli* expression. N-terminal Strep-II-tagged and C-terminal His6-tagged sequences were synthesized at Eurofins Genomics (Tokyo, Japan). The DNA fragments were cloned into the pET24(+) vector using the NEBuilder HiFi DNA Assembly Master Mix (New England Biolabs, MA, U.S.A.) according to the manufacturer’s instructions. The recombinant proteins were expressed in SHuffle T7 Express lysY- competent *Escherichia coli* (New England Biolabs, MA, USA) according to the manufacturer’s instructions. The cells were harvested by centrifugation and lysed by sonication in 100 mM Tris-HCl (pH 8.0) buffer containing 150 mM NaCl, 200 mM EDTA 200 mM, 1% Triton X-100, 1 mM PMSF, and 1 mg/mL lysozyme (Merck, Darmstadt, Germany). The protein was purified using Strep-Tactin®XT 4Flow® gravity flow column (IBA Lifesciences, Germany) according to the manufacturer’s instructions.

### Separation of proteins from coho salmon extract

The coho salmon (*Oncorhynchus kisutch*) meat was homogenized in PBS or in 1% NP40 lysis buffer containing 1 M Tris-HCl (pH 7.4), 10% glycerol, 137 mM NaCl, and Halt Protease Inhibitor Cocktail (Thermo Fisher Scientific, Waltham, MA, USA). After centrifugation, the supernatant was serially treated with two concentrations of ammonium sulfate; the fractions soluble in 20%, 30%, 40%, 50%, 60% ammonium sulfate but insoluble in 30%, 40%, 50%, 60%, 70% ammonium sulfate, respectively, were dissolved in deionized water. The solution was dialyzed against PBS and the protein concentration of each sample was measured using the Pierce BCA Protein Assay (Thermo Fisher Scientific). To further separate coho salmon proteins by anion exchange chromatography, five milligrams of coho salmon proteins precipitated with 50% ammonium sulfate was dissolved in 40 mL buffer A (20 mM Tris-HCl (pH 7.5)/20 mM NaCl) and loaded onto RESOURCE Q anion exchange column (GE Healthcare Life Sciences, Marlborough, MA). After elution with buffer A, proteins were eluted by a linear gradient from 0 to 40% buffer B (20 mM Tris-HCl (pH 7.5)/1M NaCl) at a flow rate of 6 mL/min to collect fractions (500 μL each one). The protein concentration of each fraction was measured using the Pierce BCA Protein Assay (Thermo Fisher Scientific). Equal amounts of protein in each fraction were separated by sodium dodecyl sulfate polyacrylamide gel electrophoresis (SDS-PAGE) and visualized using Coomassie Brilliant Blue (CBB) staining.

### Evaluation of total or specific IgE

Enzyme-linked immunosorbent assay (ELISA) kits for total IgE (eBioscience, San Diego, CA, USA) were used. Specific IgE against Der p, Der f, or coho salmon extract, and recombinant proteins of coho salmon tropomyosin or actin were determined using luminescence ELISA as previously described (15, 24, 25). BALB/c mice were intraperitoneally injected with 30 μg of Der p or Der f extract plus 2 mg alum or with PBS plus 2 mg alum three times at 1-week intervals (days 0, 7, and 14), followed by intranasal administration of 20 μg (20 μL) of Der p extract or PBS (20 μL) five times a week for two weeks (days 21-25 and 28-32), to generate Der p or Der f extract-sensitized mice or control mice, respectively. Alternatively, BALB/c mice were intraperitoneally injected with 30 μg coho salmon extract plus 2 mg alum or PBS plus 2 mg alum six times at 1-week intervals (days 0, 7, 14, 21, 28, and 35) to generate coho salmon extract-sensitized mice or control mice, respectively. ELISA plates were coated with 20 μg/mL of Der p, Der f, or coho salmon extract, recombinant coho salmon tropomyosin or actin, and blocked with a blocking buffer containing 10% bovine serum albumin before adding serial dilutions of serum samples. For competitive ELISA, serum samples were preincubated with 1 mg/mL Der p extract, coho salmon extract, recombinant coho salmon tropomyosin, or PBS. After the plate incubation, a biotinylated anti-IgE antibody (R35-118) (BD Pharmingen, San Diego, CA, USA) and 3,3’,5,5’-tetramethylbenzidine substrate solution were added. After adding a stop solution were added (BD Biosciences, San Jose, CA, USA), the absorbance was measured at a wavelength of 450 nm wavelength using a microplate reader (15).

### Murine model of cutaneous anaphylaxis or OAS

A murine model of cutaneous anaphylaxis was established by slightly modifying a murine model of passive cutaneous anaphylaxis (15, 26–29). Briefly, Der p extract-sensitized mice or control mice were intradermally injected with 3 μg of coho salmon extract, recombinant coho salmon tropomyosin, or recombinant coho salmon actin in the ears immediately before intravenous injection of 0. 2 mL of 0.1% Evans blue dye. Thirty minutes later, the ears were removed to evaluate the amount of extravasated dye. Alternatively, the frequency of sneezing, which is the major symptom of allergic rhinitis in murine models, was analyzed for 10 min after the last challenge with Der p extract or PBS in Der p extract-sensitized mice or control mice, respectively (30, 31). A murine model of oral allergy was established as previously described (15). Der p extract-sensitized mice or control mice were administered an injection of 1 μg coho salmon extract inside the lower lip right immediately before intravenous injection of 0. 2 mL of 0.1% Evans blue dye. Thirty minutes later, the skin on the neck was removed to evaluate the amount of extravasated dye. In both models, the removed tissue was cut into small pieces that were incubated in 0.3 mL of 1N KOH overnight at 37°C with shaking. Then, 0.15 mL of 1N phosphoric acid and 0.39 mL of acetone were added to the solution. After mixing by inversion and centrifugation at 700 g for 15 min, 0.2 mL of the supernatant was added to a 96-well microplate. The absorbance was measured at 620 nm using a 96-well microplate luminometer to evaluate the amount of extravasated dye (15, 26–29).

### Histological analyses

Histological analyses were performed as described previously (15). Sections of ear skin were stained with toluidine blue or chloroacetate esterase to calculate the percentage of degranulated mast cells among the total mast cells in ear skin.

### Statistical analyses

The results are expressed as means ± standard deviation (SD). The Mann-Whitney test results are shown in **Figures 1B, C**, **2B, D**, **3D**, **4B–D, F**. The results of the paired t-test are shown in **Figure 2C**. The Wilcoxon matched-pairs signed-rank test results are shown in **Figure 2E**. The results of a paired t-test with Welch’s correction are shown in **Figures 3B** (right panel) and **6D** (left panel). The results of Brown-Forsythe and Welch analysis of variance (ANOVA) tests With Dunnett’s T3 multiple comparisons test are shown in **Figures 3B** (left and middle panels), **5A**, **6B, D** (right panel). The results of two-way ANOVA with Sidak’s multiple comparisons test are shown in **Figure 6A**. Differences between groups were compared, and *p < 0.05 or **p < 0.01 was considered statistically significant.

## Results

### Identification of foods that may cross-react with Der p extract

To identify foods that may cross-react with the Der p extract, we used a murine model which we had recently established (15). To this end, BALB/c mice were intraperitoneally injected with Der p extract plus alum or PBS plus alum as a control six times at 1-week intervals (days 0, 7, 14, 21, 28, and 35). Serum levels of total IgE and Der p extract-specific IgE on day 42 after the sixth injection of Der p extract plus alum were significantly higher than those after the injection of alum alone (**Figures 1A–C**). We then conducted allergenic protein microarray analyses using serum from the mice after the sixth injection with Der p extract plus alum or PBS plus alum to analyze the binding affinity of serum IgE to 1178 types of crude protein extracts deriving from a variety of plants (vegetables and fruits), animals, processed foods (fish, shellfish, meats, eggs, insects, and ticks), and microorganisms (bacteria and fungi). The relative binding affinities to each protein extract of serum IgE from Der p extract-sensitized mice versus control mice were calculated. The top 22 protein extracts, that were highly bound to serum IgE from Der p extract-sensitized mice, are shown (**Table 1**). It seemed reasonable to select the edible animals or plants to further analyze out of top around 20 protein extracts. We found that two protein extracts, ranked fourteenth and sixteenth, were derived from house dust mite Der p. Ten protein extracts, including those ranked first, were derived from bacteria and fungi, among which seven were from *Escherichia coli*, suggesting that these microorganism-derived proteins may be included in the Der p extract. Notably, the second, seventh, and twenty-second-ranked species were derived from *Oncorhynchus kisutch* (coho salmon), a member of the *Salmonidae* family. The extract from coho salmon among fish tested, including *Scomber japonicus* (Chub mackerel), *Gadus macrocephalus* (Pacific cod), *Sardinops melanostictus* (sardine), and *Thunnes orientalis* (Pacific bluefin tuna), was most highly bound to serum IgE from Der p extract-sensitized mice. The twelfth-ranked was a mixture of several types of shellfish. The eighteenth and twenty-first-ranked were from *Sus scrofa domesticus* (pork) and *Gallus gallus domesticus* (chicken), respectively. Here, we focused on *Oncorhynchus kisutch* (coho salmon), an edible fish that may contain proteins that cross-react with Der p allergens.

**Table 1:** Relative binding intensity of serum IgE to the respective protein extracts.

	ID	Classification-1	Classification-2	Allergen	Intensity
1	000094	Bacteria	*Enterobacteriaceae*	*Escherichia coli serotype O45:H2*	1.79
2	001274	fish and shellfish	*Salmonidae*	*Oncorhynchus kisutch* (coho salmon)	1.71
3	000505	Plant	*Calophyllaceae*	*Calophyllum inophyllum.*	1.67
4	000098	Bacteria	*Enterobacteriaceae*	*Escherichia coli serotype O111:H8*	1.57
5	000849	Plant	*Lamiaceae*	*Salvia officinalis*	1.52
6	000097	Bacteria	*Enterobacteriaceae*	*Escherichia coli serotype O104:H12*	1.42
7	000564	fish and shellfish	*Salmonidae*	*Oncorhynchus kisutch* (coho salmon)	1.41
8	000113	Bacteria	*Streptococcaceae*	*Streptococcus pyogenes*	1.32
9	000957	Fungus	*Aureobasidium*	*Aureobasidium pullulans*	1.32
10	000072	Plant	*Cupressaceae*	*Chamaecyparis obtusa*	1.25
11	000095	Bacteria	*Enterobacteriaceae*	*Escherichia coli serotype O91*	1.16
12	000817	fish and shellfish	Shellfish Mix	clam, crab, oyster, scallops, and shrimp	1.15
13	000110	Bacteria	*Salmonella enterica*	*Salmonella Typhimurium*	1.08
14	000031	Mite	*Dermatophagoidinae*	*Dermatophagoides pteronyssinus*	1.07
15	000104	Bacteria	*Enterobacteriaceae*	*Escherichia coli serotype O145:H2*	1.04
16	000086	mite	*Dermatophagoidinae*	*Dermatophagoides pteronyssinus*	0.95
17	000105	bacteria	*Enterobacteriaceae*	*Escherichia coli serotype O157:H7*	0.92
18	000623	animal	*Suidae*	*Sus scrofa domesticus* (pork)	0.88
19	000103	bacteria	*Enterobacteriaceae*	*Escherichia coli serotype O121:H19*	0.88
20	000111	bacteria	*Shigella*	*Shigella*	0.88
21	000652	animal	*Phasianidae*	*Gallus gallus domesticus* (chicken)	0.84
22	000565	fish and shellfish	*Salmonidae*	*Oncorhynchus kisutch* (coho salmon)	0.82

The relative binding intensity of serum IgE from Der p-sensitized mice to the respective protein extracts (ID, Classification-1, Classification-2, and Allergen) was estimated by subtracting the IgE binding intensity of serum from mice injected with PBS plus alum from the IgE binding intensity of serum from mice injected with Der p extract plus alum. The shellfish mix included a mixture of clams, crabs, oysters, scallops, and shrimp.

### Serum IgE from mice sensitized with Der p extract was significantly bound to coho salmon extract

To test whether Der p extract showed IgE cross-reactivity with the coho salmon extract, mice were intraperitoneally injected with Der p extract plus alum or with alum alone three times at 1-week intervals, followed by intranasal administration of Der p extract or PBS, respectively, five times a week for two weeks (**Figure 2A**). On day 35, the mice administered the Der p extract exhibited higher levels of specific IgE against the Der p extract in serum compared to control mice that had not been administered the same extract (**Figure 2B**). The high levels of serum IgE binding to Der p extract were profoundly lowered by pre-incubating these sera with Der p extract before ELISA, confirming that serum IgE from Der p extract-administered mice was specifically bound to the Der p extract (**Figure 2C**). It should be noted that the mice administered Der p extract exhibited significantly higher levels of serum IgE against the self-prepared coho salmon extract than the control mice (**Figure 2D**), which were also significantly decreased by preincubation of these sera with Der p extract prior to ELISA (**Figure 2E**). These results indicated that IgE cross-reactive proteins exist between Der p and coho salmon extracts.

### Serum IgE from mice sensitized with coho salmon extract was significantly bound to Der p and Der f extract

We then tested whether the Der p or Der f extract, manufactured by a different company, also cross-reacted with the salmon extract. Similarly, Der p or Der f extract-treated mice and control mice were prepared (**Figure 3A**). The results showed that Der p or Der f extract-administered mice exhibited high levels of specific IgE against both Der p and Der f extracts in serum, while serum IgE from these mice was bound significantly to the salmon extract compared to serum from control mice (**Figure 3B**). To further verify the IgE cross-reactivity between Der p and coho salmon extract, mice were intraperitoneally injected with salmon extract plus alum or PBS plus alum as a control six times at 1-week intervals. The results showed that coho salmon extract-administered mice exhibited high levels of serum IgE against the coho salmon extract, whereas serum IgE from these mice was bound more strongly to both Der p and Der f extracts than serum IgE from control mice (**Figures 3C, D**). Taken together, these results suggested a possible IgE cross-reactivity between Der and coho salmon.

### Mice that had been sensitized intraperitoneally and intranasally with Der p extract exhibited evident dye extravasation in response to stimulation with coho salmon extract in the murine model of cutaneous anaphylaxis or OAS

To test whether IgE cross-reactivity between Der p and coho salmon extract causes allergic reaction in mice, we used the murine model of cutaneous anaphylaxis or OAS. Mice were intraperitoneally injected with Der p extract plus alum or alum alone three times at 1-week intervals, followed by intranasal administration of Der p extract or PBS, respectively, five times a week for two weeks (**Figure 4A**). We analyzed the frequency of sneezing during 30 min after the nasal administration of Der p extract on day 32. The results showed that Der p extract-treated mice exhibited higher numbers of sneezes than control mice, indicating that the former suffered from Der p extract-mediated allergic rhinitis in murine model (**Figure 4B**). When these mice were intradermally injected in ears with coho salmon extract just prior to intravenous injection of Evans blue dye on day 35, higher amounts of dye extravasation and more frequently degranulated mast cells in the ear skin were evident in Der p extract-administered mice but not in control mice (**Figures 4C–E**). In addition, when these mice were intravenously injected with Evans blue dye after the injection of coho salmon extract inside the lower lip on day 35, we found significantly higher amounts of dye extravasation in the neck skin of Der p extract-treated mice than in control mice (**Figure 4F**). These results indicated that the challenge with coho salmon extract induced mast cell-dependent anaphylactic responses in Der p extract-sensitized mice, presumably due to IgE cross-reactivity between Der p and coho salmon extract.

### The approximately 38-kDa protein present in the coho salmon extract was possibly bound to serum IgE from Der p extract-sensitized mice

To identify the IgE cross-reactive proteins between Der p and coho salmon extracts, coho salmon extract proteins were separated by ammonium sulfate precipitation. Notably, coho salmon proteins that had not been precipitated with 40% ammonium sulfate but precipitated with 50% ammonium sulfate were bound most highly by serum IgE from both Der p and Der f extract-sensitized mice as compared with serum IgE from control mice. Serum IgE from both Der p and Der f extract-sensitized mice also bound to coho salmon proteins that had not been precipitated with 50% ammonium sulfate but precipitated with 60% ammonium sulfate (**Figure 5A**). Then, coho salmon proteins precipitated with 50% ammonium sulfate were subjected to anion exchange chromatography to separate proteins based on their net surface charge, showing that proteins were abundantly included in fractions A2 to A3 and C7 to C14 (**Figure 5B**). Equivalent amounts of total protein in each fraction were subjected to SDS-PAGE, and the protein bands were stained with CBB. We found approximately 34 kDa or 38 kDa proteins as the major protein bands in the A2–A3 fractions or C7–C14 fractions, respectively (**Figure 5C**). In addition, we examined the binding capacity of serum IgE from Der p-sensitized mice and control mice to wells coated with equal amounts of total protein from each fraction. The results showed that serum IgE from Der p-sensitized mice was bound strongly to the proteins included in the C7–C14 fractions compared to serum IgE from control mice (**Figure 5D**). These results suggest that the approximately 38 kDa protein of coho salmon in fractions C7 to C14 was strongly bound by serum IgE from Der p extract-sensitized mice.

### Tropomyosin was identified as the IgE cross-reactive protein between Der p and coho salmon extract

To identify the approximately 38 kDa protein, that was bound strongly to serum IgE from Der p extract-sensitized mice, we performed N-terminal amino acid sequence analysis of an approximately 38 kDa band using the Edman degradation method; however, we could not do so, presumably because of the chemically modified N-terminus of this protein. Instead, we considered tropomyosin as a candidate coho salmon protein of approximately 38 kDa, because tropomyosin is known to show IgE cross-reactivity between house dust mites and crustaceans (16–19). After producing, recombinant proteins of coho salmon tropomyosin and coho salmon actin as a control, we tested whether serum from Der p extract-sensitized mice was bound to recombinant coho salmon tropomyosin or actin. The results showed that the recombinant coho salmon tropomyosin protein, but not coho salmon actin, was strongly bound by the serum IgE from Der p extract-sensitized mice (**Figure 6A**). In addition, the binding of serum IgE from Der p extract-sensitized mice to coho salmon extract or coho salmon proteins precipitated with 50% ammonium sulfate was substantially inhibited by serum preincubation with coho salmon tropomyosin (**Figure 6B**). According to UniProt sequences, tropomyosin of American house dust mite (Der p 10) shares 98.2%, 82.0%, and 50.6% amino acid sequence identity with tropomyosin of European house dust mite (Der f 10), tropomyosin 1 of *Homarus americanus* (American lobster), and tropomyosin 1 of *Oncorhynchus kisutch* (coho salmon), respectively (**Figure 6C**). These results indicate that tropomyosin is one of the major proteins responsible for IgE cross-reactivity between house dust mite Der and coho salmon. It should be noted that Der p 10 shares 75.4% amino acid sequence identity with Tropomyosin α-1 of *Mus musculus* (mouse) and Tropomyosin α-1 of *Homo sapiens* (human) (**Figure 6C**). To finally verify this IgE cross-reactivity *in vivo*, Der p extract-sensitized mice or control mice were intradermally injected with coho salmon proteins precipitated with 50% ammonium sulfate or coho salmon tropomyosin in ears before intravenous injection of Evans blue dye. Der p extract-sensitized mice exhibited higher amounts of dye extravasation than the control mice (**Figure 6D**). However, Der p extract-sensitized coho salmon actin-stimulated mice did not exhibit evident dye extravasation in their ears (**Figure 6D**). Taken together, our murine model identified tropomyosin as an IgE cross-reactive protein between house dust mite Der p and coho salmon.

## Discussion

Many questions remain regarding the development of food allergies; however, IgE cross-reactivity between environmental allergens may partly explain the mechanisms underlying food allergies subsequent to other allergic diseases, including atopic dermatitis, allergic rhinitis, allergic conjunctivitis, and allergic asthma (1–8). Recent studies indicate that exposure of food allergens on skin with barrier dysfunction strongly induces IgE sensitization, thereby causing food allergic responses following intake of the same allergens (32–34). However, sensitization to environmental allergens (e.g., pollen and HDM allergens) through various organs (e.g., the nose, eyes, lungs, and skin) may be responsible for food-related allergic responses after the intake of specific foods containing IgE cross-reactive allergens. This is also the case for PFAS (9–15). When IgE cross-reactive allergens are present in different foods, it is possible that an allergy to a specific food will subsequently cause another food allergy. This may be the case in patients with allergies to multiple, seemingly irrelevant foods. Nonetheless, IgE cross-reactivity between environmental allergens is not fully understood. Therefore, we attempted to identify food allergens that might cross-react with HDM allergens, which are known to trigger several allergic diseases, by improving a previously developed method using a murine model of sensitization and allergic protein microarray technology (15, 23). Analysis of Der p extract-sensitized mice versus control mice using allergic protein microarray technology identified *Oncorhynchus kisutch* (coho salmon) as a food containing allergens cross-reactive with Der p. In fact, serum IgE from Der p extract-sensitized mice was bound to the commercially available coho salmon extract, whereas serum IgE from coho salmon extract-sensitized mice was bound to the Der p extract. Importantly, administration of coho salmon extract induced an increase in local vascular permeability through IgE-mediated mast cell degranulation in Der p extract-sensitized mice, but not in control mice, in murine models of both cutaneous anaphylaxis and OAS (15). These *in vitro* and *in vivo* results corroborated the IgE cross-reactivity between Der p and coho salmon extract. Surprisingly, serum IgE from Der p extract-sensitized mice was preferentially bound to the extracts derived from *Escherichia coli*, which was not observed in a previous study using serum IgE from ragweed pollen-sensitized mice (15). We speculate that proteins from *Escherichia coli* may be present in the Der p extract; that is, *Escherichia coli* exists in the microbiome of HDM. Accordingly, it may be speculated that exposures to HDM may induce the production of IgG/IgE against *Escherichia coli*, that requires further verification. On the other hand, when using serum from mice sensitized with crude extracts from animals or plants in our model, we need to keep in mind that their microbiome may influence the protein microarray data. Alternatively, microarray plates are coated with various crude protein extracts. Each sample has a variety of allergens with different quantities and stabilities, which can be affected by cultivar, climates, or chemical and heat treatments (15). Therefore, the part and condition of each plant or animal sample used and the procedures of protein extraction can also impact the protein microarray data. In any case, we need to carefully analyze the protein microarray data after taking these factors into consideration.

Importantly, the approximately 38 kDa protein present in the coho salmon extract was identified as one of the major IgE cross-reactive proteins between Der p and coho salmon extracts. This finding was guided by several experiments, including protein separation from coho salmon extract by ammonium sulfate precipitation and anion exchange chromatography, visualization of proteins separated by SDS-PAGE, and measurement of the binding affinity of serum IgE from Der p extract-sensitized mice versus control mice to separate protein fraction-coated plates by ELISA. According to recent reports, salmon tropomyosin is an allergenic protein with approximately 38 kDa molecular weight. In addition, IgE cross-reactivity between HDM tropomyosin (e.g., Der p 10 and Der f 10) and shrimp tropomyosin is often responsible for shrimp allergy found in patients with HDM allergies. Accordingly, it seems plausible that tropomyosin acts as an IgE cross-reactive protein between Der p and salmon extracts. Preincubation with coho salmon tropomyosin strongly suppressed the binding of serum IgE from Der p-sensitized mice to the plate-coated coho salmon extract. Moreover, the administration of coho salmon tropomyosin induced a local anaphylactic response in Der p-sensitized mice. Although N-terminal amino acid sequencing of the corresponding band using the Edman degradation method was unsuccessful in this case, this method and/or protein mass spectrometry will be useful for identifying unknown proteins of interest included in the condensed fraction.

Because crustaceans (e.g., shrimp and crab) and arachnids (e.g., HDM) are arthropods, it is reasonable that IgE cross-reactive tropomyosin between crustaceans and HDM plays an important role in crustacean allergy (16–19). Serum IgE from Der p-sensitized mice was bound strongly to a mixture of clams, crabs, oysters, scallops, and shrimp. Although a recent report has pointed to the significance of tropomyosin as a fish allergen (20–22), our murine model showed a possible causal relationship between HDM and salmon allergies. It may be speculated that patients with HDM allergy develop salmon allergy; however, further clinical investigation is required to clarify the significance of this relationship. Interestingly, protein microarray analysis showed that the extract from coho salmon among fish tested was most highly bound to serum IgE from Der p extract-sensitized mice. On the other hand, American house dust mite tropomyosin shares 82.0%, 75.4% or 50.6% amino acid sequence identity with American lobster tropomyosin 1, mouse and human Tropomyosin α-1, and coho salmon tropomyosin 1, respectively. Accordingly, one possible explanation is that tropomyosin from coho salmon among tropomyosin-containing lives may contain conformational IgE epitope similar to arthropod tropomyosin. In any case, further examination will be required to completely understand the relevant molecular mechanisms.

In conclusion, our murine model identified tropomyosin as an IgE cross-reactive protein between HDM and coho salmon, illustrating salmon allergy following HDM allergy. Our method for identifying IgE cross-reactive allergens will help clarify the unknown mechanisms underlying the development of food allergies.

## Data availability statement

The datasets presented in this study can be found in online repositories. The names of the repository/repositories and accession number(s) can be found in the article/supplementary material.

## Ethics statement

All the procedures were approved by the Institutional Review Committee of Juntendo University (approval numbers: 2021188, 2022099, and 2023129). The study was conducted in accordance with the local legislation and institutional requirements.

## Author contributions

RY performed all the experiments and participated in writing the manuscript. KI assisted with the analysis of murine model and the *in vitro* experiments, analyzed the data, and actively participated in manuscript writing. TA assisted with the *in vitro* experiments and statistical analysis and analyzed the data. AT assisted with protein purification. ST, AM, HW, MN, and NNe assisted with the *in vitro* experiments. AK, HY, SU, AY, YK, and NNa assisted with the *in vivo* experiments. NE, TS, HO, and KO analyzed the data. JK conceived the project, analyzed the data, and actively participated in manuscript writing. All authors contributed to the article and approved the submitted version.
